# Risk analysis and outcome of mediastinal wound and deep mediastinal wound infections with specific emphasis to omental transposition

**DOI:** 10.1186/1749-8090-6-111

**Published:** 2011-09-19

**Authors:** Haralabos Parissis, Bassel Al-Alao, Alan Soo, David Orr, Vincent Young

**Affiliations:** 1 Cardiothoracic Dept, Royal Victoria Hospital, Grosvenor Rd, Belfast, BT12 6BA, UK; 2 Plastic Surgery Dept, St James Hospital, St James Street, Dublin, Dublin 8, Republic of Ireland; 3 Cardiothoracic Dept, St James Hospital, St James Street, Dublin, Dublin 8, Republic of Ireland

## Abstract

**Background:**

To report our experience, with Deep mediastinal wound infections (DMWI). Emphasis was given to the management of deep infections with omental flaps

**Methods:**

From February 2000 to October 2007, out of 3896 cardiac surgery patients (prospective data collection) 120 pts (3.02%) developed sternal wound infections. There were 104 males & 16 females; (73.7%) CABG, (13.5%) Valves & (9.32%) CABG and Valve.

**Results:**

Superficial sternal wound infection detected in 68 patients (1.75%) and fifty-two patients (1.34%) developed DMWI. The incremental risk factors for development of DMWI were: Diabetes (OR = 3.62, CI = 1.2-10.98), Pre Op Creatinine > 200 μmol/l (OR = 3.33, CI = 1.14-9.7) and Prolong ventilation (OR = 4.16, CI = 1.73-9.98). Overall mortality for the DMWI was 9.3% and the specific mortality of the omental flap group was 8.3%. 19% of the "DMWI group", developed complications: hematoma 6%, partial flap loss 3.0%, wound dehiscence 5.3%. Mean Hospital Stay: 59 ± 21.5 days.

**Conclusion:**

Post cardiac surgery sternal wound complications remain challenging. The role of multidisciplinary approach is fundamental, as is the importance of an aggressive early wound exploration especially for deep sternal infections.

## Introduction

The incidence of mediastinal wound infection in patients undergoing median sternotomy and open-heart surgery can be up to 5%[1], [2]. A subgroup of 20-30% of those patients [3] develops deep sternal infections with an associated morbidity, mortality, and "cost" that remain unacceptably high [4]. There is a considerable lack of consensus regarding the ideal operative treatment of complicated (class 2b) El Oakley [5] sternal wounds. The initial treatment with open packing and antibiotic irrigation carries high mortality (up to 50% at Emory series) [6] and has become the treatment of the past. Current treatment with radical sternal debridement and closure using muscle or omental flaps has become popular and is possibly associated with lower mortality. This paper reports our experience on the management of mediastinal wound infections with specific focus on the use of omental flaps.

## Methods

From February 2000 to October 2007, 3896 patients underwent open heart surgery. Prospective data acquisition pertained to the patients was based upon the dataset defined by the Society for Cardiothoracic Surgery in Great Britain and Ireland.

Superficial sternal wound infection was defined as sternal discharge confined to the skin and subcutaneous tissues with no sternal instability. The presence of sepsis associated with sternal instability, purulent discharge and positive microbiology, defined deep mediastinal wound infections. Non-infected, "mechanical" dehiscence's (El Oakley class I) were excluded from this study.

Collection of the data is served using the Patients Analysis and Tracking System (PATS) software. Eighty variables were prospectively collected and carefully validated before being analyzed.

Categorical variables were tested using a qui square test or Fisher exact test (two-tailed), and continuous variables were tested using Students t test (two-tailed). A p Value of less than 0.05 was regarded as statistical significant. All calculations were made using SPSS 11 edition. Operative mortality is reported as 30-day mortality, or as mortality occurred during the same hospital admission (when the hospital stay was more than 30 days).

*Bilateral pectoralis major myocutaneous advancement flap with greater omental transposition: Surgical technique *(*See *Figures 1, 2, 3, 4, 5, 6, 7 and 8)

**Figure 1:**
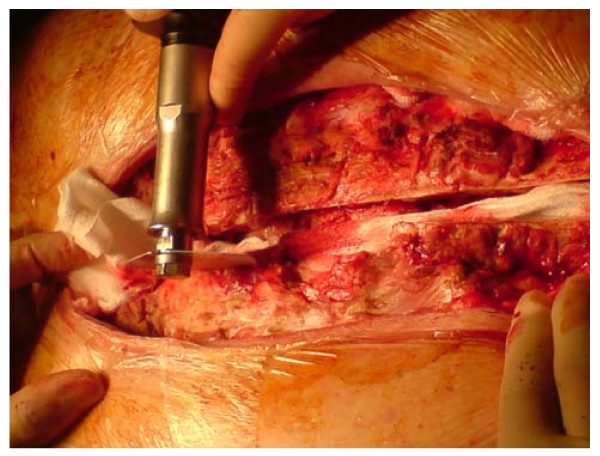
**Extensive bone debridement with a redo saw**.

**Figure 2:**
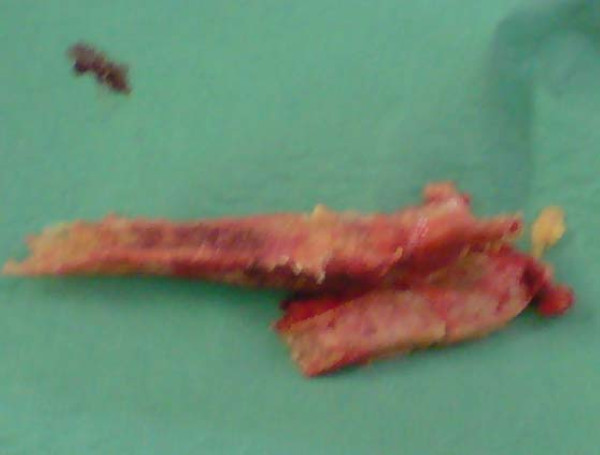
**Sternal excision**.

**Figure 3:**
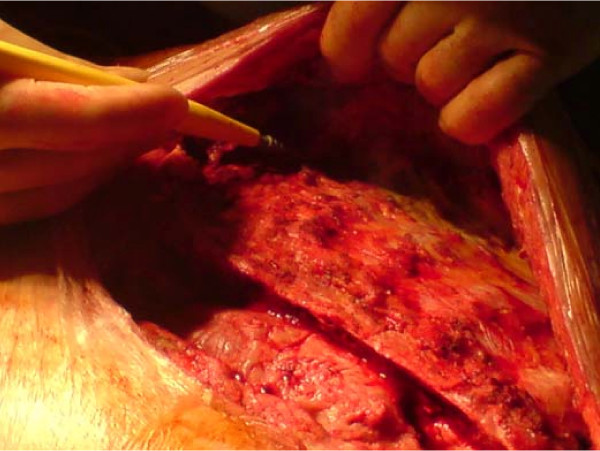
**Raising of the pectoral flaps, by detaching the pectoral muscle, off the chest wall**.

**Figure 4:**
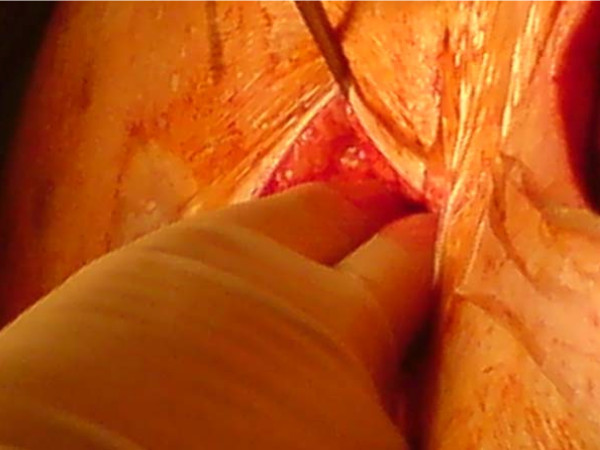
**Opening of the abdomen for the harvesting of an omental flap**.

**Figure 5:**
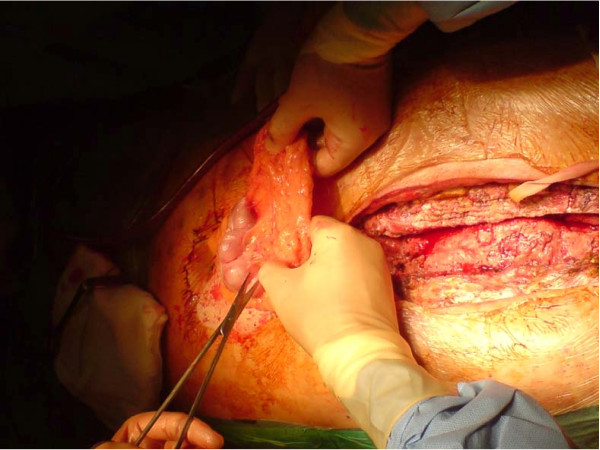
**Harvesting of the in situ omental flap**.

**Figure 6:**
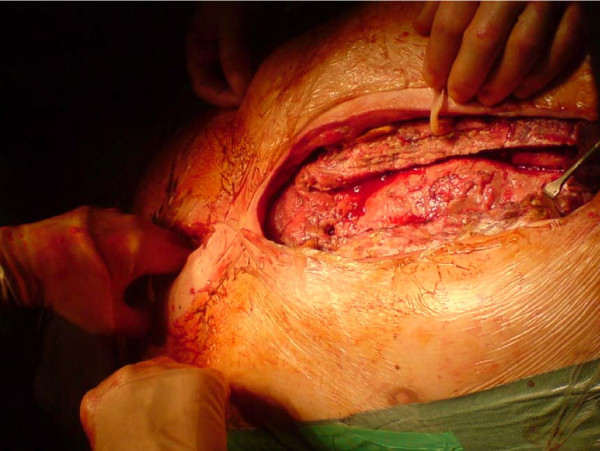
**Coverage of the anterior mediastinum with omentum, by transferring the omental graft via an anterior opening of the diaphragm**.

**Figure 7:**
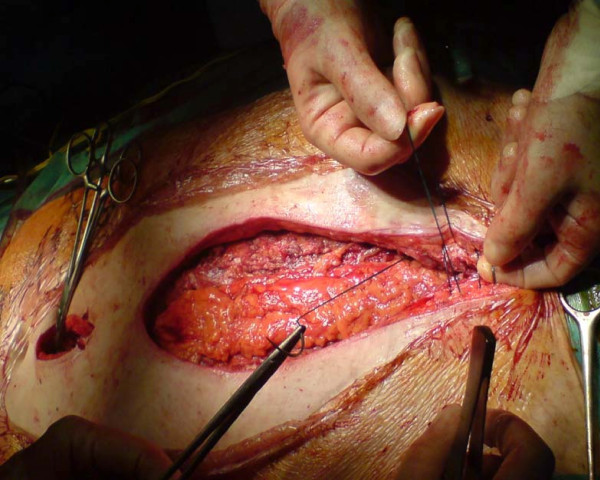
**The omental flap is covered the anterior mediastinum**. The pectoral muscle is approximated in the middle line using nylon loops. We avoid undermining the Pectoralis muscle off the subcutaneous tissues and that preserves blood supply in the area.

**Figure 8:**
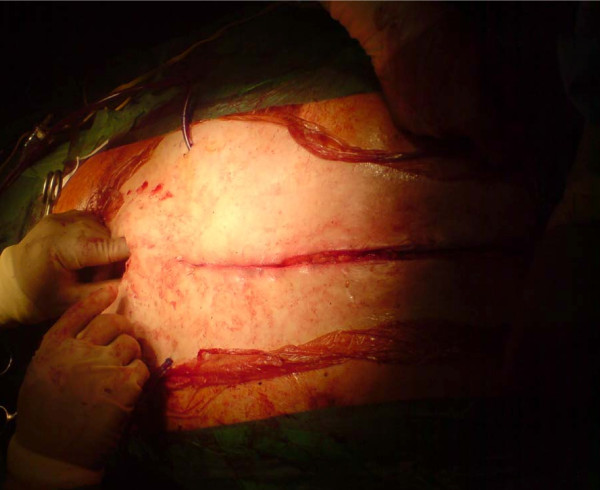
**The end result**.

The omentum, a well vascularised tissue with its immunologic and angiogenic properties, is a versatile organ with well-documented utility in the reconstruction of complex wounds and defects. In our series it was used as a pedicle. The median sternotomy incision is only extended for 2 inches towards the umbilicus and the peritoneal cavity is entered. The omentum is mobilized and is brought up in to the chest through a diaphragmatic opening; it fills the gap of the missing sternum quite adequately. The pectoralis major muscle based on the thoracoacromial artery is also mobilized. This facilitates apposition of the pectoral musculature and subcutaneous tissue "en mass" on top of the omentum, in the middle line. We specifically avoid undermining the Pectoralis muscle off the subcutaneous tissues and that preserves blood supply.

### VAC pump

Vacuum-assisted closure system consisting of polyurethane foam pieces and a special pump unit was used. The foam was placed in the wound after debridement of foreign material and necrotic tissue. The wound was covered with adhesive drape and connected to the pump unit, which was programmed to create a continuous negative pressure of 125 mm Hg in the wound cavity.

## Results

Out of 3896 patients, 120 patients (3.02%) developed sternal wound infections; There were 104 males and 16 females. 89 patients had undergone CABG (73.7%), 16 patients had Valve Surgery (13.5%), 11 patients had CABG and Valves (9.17%) and 4 patients (3.3%) had various procedures. Overall, sternal wound infections were diagnosed in 3.34% of the CABG patients, 3.79% of the CABG and Valves and 3% of the Valve patients. Patient's demographics are presented in Table 1. The overall mortality of the patients that they developed sternal wound infections was 9.16% (11 patients). Concomitant leg wound infection was found in 13 patients (10.84%). Sixty-eight patients (1.75%) developed superficial sternal wound infection and treated with appropriate antibiotics, local drainage and debridement of the wound. The mortality of this group was 4.41% (3 patients).

**Table 1:** Patient Characteristics

Patient Demographics	Superficial N = 68	DMWIN = 52	Control N = 3896	pValue
Age	66.3 ± 9.9	67.1 ± 8.7	63.7 ± 10.7	NS
Gender (M)	86.7%	84.5%	78%	NS
DM	19.2%	28.8%	Diet: 4.5% Oral:8% Insulin:3.6%	0.023
Creatinine > 200 μmol/l	1.95%	5.05%	2.21%	0.027
Smoking History	67.8%	78.9%	69.7%	NS
PVD	14.6%	16.8%	18.3%	NS
COAD	13.2%	19.2%	17.9%	NS
Leg wound infection	10.3%	11.5%	9.1%	NS
BMI > 30	44.2%	46.1%	42.9%	NS
EF	Good: 63.7% Moderate: 31.4% Poor: 4.9%	Good: 65.4% Moderate 28.5% Poor: 6.1%	Good: 68.4% Moderate: 26.3% Poor: 5.3%	NS
Priority (Elective)	33.8%	32.7%	34.5%	NS
Logistic Euroscore	4.2 ± 1.9	7.3 ± 3.6	3.71 ± 1.25	NS
Reoperation for bleeding	4.4%	3.9%	4.5%	NS
Prolong ventilation	5.8%	34.6%	6.8%	< 0.001
Tracheostomy	1.5%	9.6%	1.8%	< 0.001
New dialysis required	4.4%	11.5%	4.9%	< 0.001
Re-intubation rate	4.4%	13.4%	4.7%	< 0.001
Hospital stay(days)	19 ± 6	59 ± 21.5	9 ± 2.5	< 0.001

### The DMWI group

Fifty two patients (1.34%) developed DMWI. The overall mortality of this group was 15.38% (8 patients).

#### The microbiology of the patients with DMWI

Blood cultures were positive in 30% of the patients with DMWI. Wound microbiology revealed S. aureus (32%), Coagulase Negative Staphylococcus (29.6%), methicillin-resistant Staphylococcus aureus (MRSA) (2.3%), Vancomycin Resistant Enterococcus (VRE) (3.8%), Cram negative (17.5%) & other 14.8% (Anaerobics 1.2%, Fungal 4%).

The incremental risk factors (see Table 2) for development of DMWI were: Diabetes (OR = 3.62, CI = 1.2-10.98), pre-operative Creatinine > 200 μmol/l (OR = 3.33, CI = 1.14-9.7) and prolong ventilation (OR = 4.16, CI = 1.73-9.98). Complications were developed in 9 patients (17.3%): Seroma-hematoma 5 patients (9.62%), partial flap loss 2 patients (3.85%), wound dehiscence 2 patients (3.85%). Mean Hospital Stay: 59 ± 21.5 days. The likelihood of developing complications in patients with DMWI was higher: re-intubation rate 13.4%, new dialysis required 11.5%, Tracheostomy 9.6%, Prolong ventilation 34.6%. All the patients with DMWI had their wounds checked at 6 months and 1 year following discharge. Healed wounds: 50 patients (96.2%), persistent pain and discomfort: 19 patients (37%), paresthesia-numbness 16 patients (30.7%) and feeling of "sternal instability" 20 patients (38.5%).

**Table 2:** Multivariate logistic regression analysis of the risk factors influencing DMWI

		O.R.	95% C.I.		
Risk factor					p value
Diabetes	Non	1.00			
	DMWI	3.62	1.20	10.98	0.023
					
Pre Op Creatinine > 200 μmol/l	Non	1.00			
	DMWI	3.33	1.14	9.70	0.027
					
Prolong ventilation	Non	1.00			
	DMWI	4.16	1.73	9.98	0.001

### VAC pump Group

18 patients (0.47%) were treated with vacuum assisted closure VAC pump and secondary wound closure, due to a partial sternal instability. There were initial treatment failures in 2 patients requiring surgical revision. The mortality for this group was 11.11% (2 patients).

### Sternal debridement & primary re-suturing

16 patients (0.41%) were treated with early sternal wound revision. In this group of patients during the early post operative period the sternum became unstable and purulent discharge was detected. The wound was reopened, and the sternum was debrided; primary rewiring was deemed suitable because the sternal bone was at least partially intact. A betadine or Vancomycin irrigation system was placed in situ. The overlying musculocutaneous tissue was closed over deep tension sutures. Eventually the irrigation was removed when 3 negative microbiology specimens were detected from the efflux fluid. This group, consist off, males. Five (5) patients had CABG, five (5) CABG & Valve and one (1) patient has had Valve and other. There were initial treatment failures in 3 patients, which led to revisions. The mortality of this group was 18.75% (3 patients).

### DMWI treated with Flaps

18 patients (0.47%) had various flaps; 12 omental, 3 combination of rectal abdominal and pectoral flaps and 3 solely pectoral flaps. All the omental flaps were performed following initial application of VAC pump up till the purulent infection settled. There were 16 males. The mean Euroscore of this group was 5.8 (ranges, between 1-13). Ten (10) patients had CABG, six (6) had Valves and two (2) had CABG and Valve. The mean Intensive Care Unit stay was 21.2 days (ranges, between 4 to 60 days). Two (2) patients developed post-operative sepsis requiring inotrops and in two (2) patients Vancomycin Resistant Enterococcus (VRE) was isolated. There were initial treatment failures in 1 patient, who required operative revision and eventually closure of the wound with the aid of a VAC-pump. The mortality of this group was 16.66% (3 patients).

## Discussion

Radical debridement in order to eradicate infection is of a paramount importance therefore sternal excision becomes a necessity in cases with severe sternal involvement. Under those circumstances various flaps have been used; this study is not comparing the various treatment strategies for DMWI because the number of the patients involved is small, however outlines a trend of action and also emphasizes the technique of omental flap use.

The surgical approach for the treatment of DMWI varies according to surgeon preference due to lack of robust clinical evidence. A more favorable outcome has been linked to different treatment strategies. Evolution in treatments has led from tube irrigation of the mediastinum to the use of negative pressure wound therapy VAC pump [7] and lately to the introduction of muscle flap coverage.

We agreeably accept that there is a role for all those therapeutic modalities. During early diagnosis, of DMWI with a salvageable sternum we advocate reopening of the wound, debridement and rewiring. Tube irrigation of the mediastinum using betadine or vancomycin infusion is installed. The wound is close primarily with tension sutures; however, if the subcutaneous tissues are under tension we use advanced pectoral flaps.

**Figure 9:**
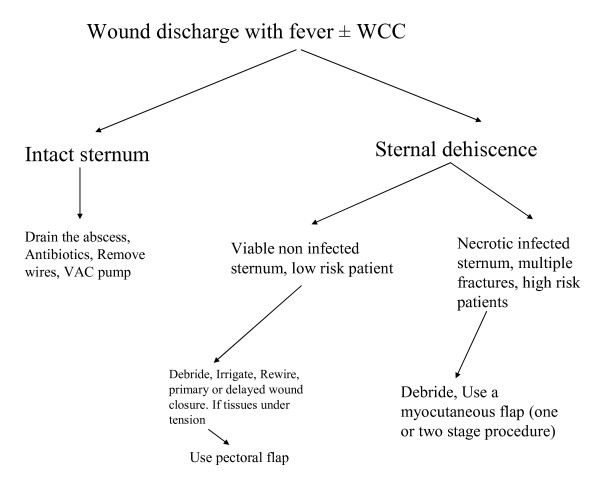
**Algorithm for the management of sternal wound infections**.

When the sternum is fractured in multiple places in a high-risk patient (Severe COAD, use of BIMAs, alcocholism, renal impairment, steroid therapy, and previous radiation to the chest) or there is sternal ostomyelitis then we excise the bone and fill the gap with omentum. The wound is closed over advanced pectoral flaps (The algorithm for the management of sternal wound infections is presented in Figure 9). The latest strategy can be performed in two ways: 1) For uncontrolled mediastinal sepsis, serial debridement and VAC pump with delayed omental flap transposition and 2) single-stage management, which consisted of debridement of the sternal wound and omental flap transposition. The need for laparotomy during omental harvesting and the potential for intraabdominal complications have been criticized; however donor-site complications are usually limited to abdominal wall infection and hernia [8]. Moreover, debridement and flap coverage without osseous closure makes subsequent re -interventions challenging. The loss of sternal integrity is a disadvantage, not only because in up to 40% of the patients it gives local symptoms but particularly due to the fact that makes redo operations difficult. Therefore some groups advocate thorough debridement and the use of the vacuum-assisted closure system (VAC pump) for few weeks following by the use of sternal clips [9] or sternal osteosynthesis with horizontal titanium plates that can be inserted in the parasternal space with consecutive proper stabilization of the sternum [10]. Sternal preservation whenever possible should be the aim, however if delayed diagnosis or as per Immer et al [11] mediastinitis, in old sick patients with poor vascularised multifractured sternum should be treated with sternal excision and a musculo-cutaneous flap. Prolong antibiotic treatment up to 6 weeks is usually advocated [12].

Some institutions are routinely managing deep sternal infection with sternal wound debridement, rewiring, and closed drainage, with or without antibiotic saline tube irrigation (the traditional approach). The mortality from this traditional approach could be up to 37.5% [13] until sternal debridement with muscle or omental flap reconstruction became the standard treatment for this postoperative complication and lowered the mortality rate to just more than 5% [11,13]. The mortality in our series of patients with DMWI treated with Sternal debridement & re-suturing was 9% and with omental flaps was 8.3%. This is similar to the mortality reported by other groups [14].

In our series of 52 DMWI patients, treated with 3 different modalities, the treatment failed in 6 patients (11.5%). In 5 out of those 6 patients, MRSA or VRE had been isolated. As per Douville et al, treatment failures were detected in 18.8% of the patients following Sternal debridement & re-suturing and in 24% of the muscle flap patients [15]. Moreover partial flap loss occurred in 11.6% of the patients, with no total flap failures as per Hultman and colleagues [16]. Additional procedures for recurrent sternal wound infection were necessary in 5.1% of patients [6]. The microbiology in our group of patients correlates with other reports [14,15] and includes mainly Gram positive in up to 61.6% of the patients, interestingly however in our report MRSA and VRE was higher and up to 6.1%. It is worth mentioning, that according to Yasuura et al [17] patients with blood culture positive for methicillin-resistant Staphylococcus aureus had recurrent sternal infections. Independent predictors for DMWI in our study was diabetes, preoperative renal impairment and prolonged ventilation and ICU stay such as alcocholics following re-intubation and prolonged intensive care unit stay following delirium or prolong ventilation following a stroke. The use of BIMAs in our institution was limited; therefore we were unable to derive substantial conclusions regarding BIMAs.

A large report from Emory University [6] reported the 20 year institutional experience with 409 musculocutaneous flaps. There were: Pectoralis major flaps: 440 patients, Rectus abdominal flaps: 202 patients, Omental flaps: 16 patients. The Risk factors for developing DMWI were COAD, IABP use and the use of IMA, BIMAs. Wound complications occurred in 19%. Mortality was 8-10% and Risk factors for death were septicemia, preoperative MI, and the use of IABP.

One year follow up of our patients showed healed wounds in 50 patients (96.2%), however almost a third of the patients continue to have persistent pain and discomfort paresthesia and a feeling of "sternal instability". Long term results following sternal reconstruction were reported by Ringelman et al [18]; 99% of the wounds were healed. The morbidity however was high with persistent pain and discomfort in 50% of the cases, Paresthesia-numbness in 44%, Sternal instability in 42%, Post-operative weakness in the Shoulder-abdomen in 32%of the cases, Inability to perform the same pre-operative activities in 36% and finally Contour abnormalities of the chest and abdomen in 85% of the patients. Furthermore, Braxton et al [19] reported that Mediastinitis is associated with a marked increase in mortality during the first year post-CABG and a threefold increase during a 4-year follow-up period.

Compare to the rest of the cardiac surgical population, the subgroup of patients that developed DMWI had a similar incidence of reoperation for bleeding. However, much higher incidence of prolonged ventilation, re-intubation rate, tracheostomy rate and "new dialysis required" was encountered in those patients.

Our study supports the concept of using bilateral pectoralis major myocutaneous advancement flap with greater omental transposition in DMWI, when the sternum is not viable or if the patient is a high risk. This approach was tested in a small number of patients and was found superior according to Brandt et al [20], and Eifert et al [21]. However until level I evidence are available, clear cut indications as to who would benefit from which approach, are lacking in the literature.

The initial limitation of our study is derived by its observational retrospective nature. Our database consists of prospectively collected data; however, it was not design to prospectively compare different strategies for the treatment of DMWI. Furthermore the number of the patients examined is small and also our follow up is limited to one year.

## Conclusions

Post cardiac surgery sternal wound complications remain challenging. Efforts should focus on prevention such as better perioperative glycaemic control [22]. Unfortunately, in patients with an increased risk for sternal instability and wound infection after cardiac surgery, sternal reinforcement according to the technique described by Robicsek did not reduce this complication [23]. DMWI is associated with an increase rate of Morbidity &Mortality, as well as high costs [24]. Aggressive early wound exploration especially for DMWI and multidisciplinary approach involving plastic surgeons early in the course, is of a paramount importance.

Possibly, flap repair is superior to more conservative surgical options such as sternal resuturing with mediastinal irrigation. Further reductions in mortality will depend on earlier detection of mediastinitis, before the onset of septicemia, and ongoing multisystem organ failure.

## Competing interests

The authors declare that they have no competing interests.

## Authors' contributions

HP gathered the data, participated in the sequence alignment and drafted the manuscript, BA assist in data analysis, statistics and also the development of the manuscript, AS helped with the collection of the data and the construction of the manuscript, DO (Plastic Surgeon) participated in its design and coordination and performed the omental harvesting and surgery in the group of patients needed omental flaps and VY overlooked the progress of the manuscript and advised on valuable amendments. The authors read and approved the final manuscript.

## References

[B1] Riddlerstolpe L Gill H Granfeldt H Rutberg H Superficial and deep sternal wound complications: incidence, risk factors and mortality Eur J Cardiothorac Surg 2001 20 1168 75 10.1016/S1010-7940(01)00991-5 11717023

[B2] Olsen MA Lock-Buckley P Hopkins D Polish LB Sundt TM Fraser VJ The risk factors for deep and superficial chest surgical-site infections after coronary artery bypass graft surgery are different J Thorac Cardiovasc Surg 2002 124 1 136 45 10.1067/mtc.2002.122306 12091819

[B3] The Parisian Mediastinitis Study Group Risk factors for deep sternal wound infection after sternotomy: a prospective, multicenter study J Thorac Cardiovasc Surg 1996 111 1200 7 8642821 10.1016/s0022-5223(96)70222-2

[B4] Loop FDLytle BW Cosgrove DM Mahfood S McHenry MC Goormastic M Stewart RW Golding LA Taylor PCJ Maxwell Chamberlain memorial paper: sternal wound complications after isolated coronary artery bypass grafting: early and late mortality, morbidity and cost of care Ann Thorac Surg 1990 49 179 87 10.1016/0003-4975(90)90136-T 2306138

[B5] El Oakley RM Wright JE Postoperative mediastinitis: classification and management Ann Thorac Surg 1996 61 1030 6 10.1016/0003-4975(95)01035-1 8619682

[B6] Jones G Jurkiewicz MJ Bostwick J Wood R Bried JT Culbertson J Howell R Eaves F Carlson G Nahai F Management of the infected median sternotomy wound with muscle flaps. The Emory 20-year experience Ann Surg 1997 225 6 766 76 discussion 776-8 10.1097/00000658-199706000-00014 9230817 PMC1190886

[B7] Petzina R Hoffmann J Navasardyan A Malmsjö M Stamm C Unbehaun A Hetzer R Negative pressure wound therapy for post-sternotomy mediastinitis reduces mortality rate and sternal re-infection rate compared to conventional treatment Eur J Cardiothorac Surg 2010 38 1 110 3 10.1016/j.ejcts.2010.01.028 20171898

[B8] Hultman CS Carlson GW Losken A Jones G Culbertson J Mackay G Bostwick J Jurkiewicz MJ Utility of the omentum in the reconstruction of complex extraperitoneal wounds and defects: donor-site complications in 135 patients from 1975 to 2000 Ann Surg 2002 235 6 782 95 10.1097/00000658-200206000-00005 12035034 PMC1422507

[B9] Reiss N Schuett U Kemper M Bairaktaris A Koerfer R New method for sternal closure after vacuum-assisted therapy in deep sternal infections after cardiac surgery Ann Thorac Surg 2007 83 6 2246 7 10.1016/j.athoracsur.2006.07.077 17532448

[B10] Baillot R Cloutier D Montalin L Côté L Lellouche F Houde C Gaudreau G Voisine P Impact of deep sternal wound infection management with vacuum-assisted closure therapy followed by sternal osteosynthesis: a 15-year review of 23,499 sternotomies Eur J Cardiothorac Surg 2010 37 4 880 7 10.1016/j.ejcts.2009.09.023 19880326

[B11] Immer FF Durrer M Mühlemann KS Erni D Gahl B Carrel TP Deep sternal wound infection after cardiac surgery: modality of treatment and outcome Ann Thorac Surg 2005 80 3 957 61 10.1016/j.athoracsur.2005.03.035 16122463

[B12] Khanlari B Elzi L Estermann L Weisser M Brett W Grapow M Battegay M Widmer AF Flückiger U A rifampicin-containing antibiotic treatment improves outcome of staphylococcal deep sternal wound infections J Antimicrob Chemother 2010 65 8 1799 806 10.1093/jac/dkq182 20542908

[B13] Netscher DT Eladoumikdachi F McHugh PM Thornby J Soltero E Sternal wound debridement and muscle flap reconstruction: functional implications Ann Plast Surg 2003 51 2 115 22 discussion 123-5 10.1097/01.SAP.0000058497.92264.E2 12897511

[B14] Sachithanandan A Nanjaiah P Nightingale P Wilson IC Graham TR Rooney SJ Keogh BE Pagano D Deep sternal wound infection requiring revision surgery: impact on mid-term survival following cardiac surgery Europ J of Cardiothorac Surgery 2008 33 673 678 10.1016/j.ejcts.2008.01.002 18243720

[B15] Douville EC Asaph JW Dworkin RJ Handy JR Jr Canepa CS Grunkemeier GL Wu Y Sternal preservation: a better way to treat most sternal wound complications after cardiac surgery Ann Thorac Surg 2004 78 5 1659 64 10.1016/j.athoracsur.2004.04.082 15511452

[B16] Hultman CS Culbertson JH Jones GE Losken A Kumar AV Carlson GW Bostwick J Jurkiewicz MJ Thoracic reconstruction with the omentum: indications, complications, and results Ann Plast Surg 2001 46 3 242 9 10.1097/00000637-200103000-00007 11293514

[B17] Yasuura K Okamoto H Morita S Ogawa Y Sawazaki M Seki A Masumoto H Matsuura A Maseki T Torii S Results of omental flap transposition for deep sternal wound infection after cardiovascularsurgery Ann Surg 1998 227 3 455 9 10.1097/00000658-199803000-00019 PMC1191285 9527070

[B18] Ringelman PR Vander Kolk CA Cameron D Baumgartner WA Manson PN Long-term results of flap reconstruction in median sternotomy wound infections Plast Reconstr Surg 1994 93 6 1208 14 discussion 1215-6 10.1097/00006534-199405000-00015 8171140

[B19] Braxton JH Marrin CA McGrath PD Ross CS Morton JR Norotsky M Charlesworth DC Lahey SJ Clough RA O'Connor GT Northern New England Cardiovascular Disease Study Group Mediastinitis and long-term survival after coronary artery bypass graft surgery Ann Thorac Surg 2000 70 6 2004 7 10.1016/S0003-4975(00)01814-2 11156110

[B20] Brandt C Alvarez JM First-line treatment of deep sternal infection by a plastic surgical approach: superior results compared with conventional cardiac surgical orthodoxy Plast Reconstr Surg 2002 109 7 2231 7 10.1097/00006534-200206000-00009 12045542

[B21] Eifert S Kronschnabl S Kaczmarek I Reichart B Vicol C Omental flap for recurrent deep sternal wound infection and mediastinitis after cardiac surgery Thorac Cardiovasc Surg 2007 55 6 371 4 10.1055/s-2007-965305 17721846

[B22] Matros E Aranki SF Bayer LR McGurk S Neuwalder J Orgill DP Reduction in incidence of deep sternal wound infections: random or real? J Thorac Cardiovasc Surg 2010 139 3 680 5 10.1016/j.jtcvs.2009.10.006 20018307

[B23] Schimmer C Reents W Berneder S Eigel P Sezer O Scheld H Sahraoui K Gansera B Deppert O Rubio A Feyrer R Sauer C Elert O Leyh R Prevention of sternal dehiscence and infection in high-risk patients: a prospective randomized multicenter trial Ann Thorac Surg 2008 86 6 1897 904 10.1016/j.athoracsur.2008.08.071 19022005

[B24] Graf K Ott E Vonberg RP Kuehn C Haverich A Chaberny IF Economic aspects of deep sternal wound infections Eur J Cardiothorac Surg 2010 37 4 893 6 10.1016/j.ejcts.2009.10.005 19896860

